# Influence of Stress-Induced Senescence on the Secretome of Primary Mesenchymal Stromal Cells

**DOI:** 10.3390/biom15121734

**Published:** 2025-12-13

**Authors:** Daria Kashirina, Diana Matveeva, Mariia Ezdakova, Alexander Brzhozovskiy, Alexey Kononikhin, Ludmila Pastushkova, Irina Larina, Evgeny Nikolaev, Ludmila Buravkova, Andrey Ratushnyy

**Affiliations:** 1 Institute of Biomedical Problems, Russian Academy of Sciences, Khoroshevskoye Shosse, 76a, 123007 Moscow, Russia; matveeva.dajana@yandex.ru (D.M.); ezdakova.mi@gmail.com (M.E.); agb.imbp@gmail.com (A.B.); as.kononikhin@gmail.com (A.K.); lpastushkova@mail.ru (L.P.); irina.larina@gmail.com (I.L.); buravkova@imbp.ru (L.B.); ratushkin@mail.ru (A.R.); 2 Project Center of Omics Technologies and Advanced Mass Spectrometry, 121205 Moscow, Russia; ennikolaev@gmail.com

**Keywords:** mesenchymal stromal cells, stress-induced senescence, secretome, mass spectrometry, proteomics, SASP

## Abstract

Mesenchymal stromal cells (MSCs) are promising therapeutic agents, largely due to their capacity for self-renewal, differentiation, and immunomodulation. Importantly, these beneficial effects are frequently mediated by the MSC secretome, which contains factors with anti-inflammatory, anti-apoptotic, and pro-regenerative properties. However, cellular senescence can impair these critical functions. To identify senescence-associated changes in the MSC secretome that may regulate aging and intercellular communication, we performed a mass spectrometry-based proteomic analysis of the conditioned medium from MSCs undergoing stress-induced senescence. Our analysis confirmed the upregulation of established aging markers, such as IL-6, PAI-1, and IGFBP7. Furthermore, we identified a significant increase in lesser-known senescence-associated secretory phenotype (SASP) components, including INHBA—a known inhibitor of proliferation—and DKK3, which blocks stromal cell pluripotency. Pathway analysis revealed that stress-induced senescence broadly affected proteins involved in glycolysis, immune response, hemostasis, and the regulation of cell death and the cell cycle. These alterations are likely to negatively impact the MSC microenvironment. Interestingly, the cellular response to senescence was dualistic. Alongside detrimental SASP factors, we observed an increase in protective proteins such as annexins (ANXA1, ANXA2), antioxidants (TXN, PRDX1, PRDX6), and the heat shock protein HSPB1, which collectively defend neighboring cells from inflammation and oxidative stress. These findings underscore the complex etiology of cellular senescence and the paradoxical nature of the SASP. The obtained data also emphasize the necessity of comprehensive proteomic profiling of the MSC secretome across different aging models to harness the full therapeutic potential of MSCs and their secretomes for regenerative medicine.

## 1. Introduction

Mesenchymal stromal cells (MSCs) are a heterogeneous population of adult stem cells capable of differentiating into a variety of mesodermal-derived cells, forming bone, cartilage, fat, muscle, tendon, stromal, and neuronal cells. These properties, combined with their potent immunomodulatory functions, underpin their broad therapeutic appeal. A growing body of evidence indicates that the positive effects of MSC transplantation are predominantly paracrine, mediated by the secretion of factors that restore tissue homeostasis [1]. As a result, the MSC secretome itself has become a viable cell-free alternative to transplantation, leveraging its anti-inflammatory, anti-apoptotic, and regenerative effects for therapeutic applications [2].

The therapeutic application of the mesenchymal stromal cell (MSC) secretome and conditioned medium is a rapidly advancing field, with numerous clinical trials registered on https://clinicaltrials.gov (accessed on 3 June 2025) targeting conditions ranging from COVID-19 and ischemic stroke to knee osteoarthritis and chronic wounds. This cell-free strategy offers significant advantages over whole-cell therapy, including a superior safety profile, easier distribution, and enhanced tissue access for soluble bioactive molecules. However, a major challenge remains the in vitro production of sufficient quantities of secretome [2].

A critical factor that can compromise the efficacy and safety of this approach is cellular senescence. Senescence induces profound morphological and functional alterations in MSCs, including an enlarged, granular morphology, reduced clonogenic capacity (evidenced by decreased colony-forming units), cell cycle arrest in the G1/G0 phase, and elevated senescence-associated β-galactosidase (SA-β-gal) activity [3]. Functionally, senescent MSCs exhibit impaired differentiation potential, contributing to conditions like osteoporosis, and a reduced ability to recruit and polarize macrophages toward the anti-inflammatory M2 phenotype [4].

Therapeutically, the most critical implication of MSC senescence is the development of the senescence-associated secretory phenotype (SASP). The SASP comprises a plethora of factors—including pro-inflammatory cytokines, chemokines, growth factors, and matrix metalloproteinases—that can induce secondary senescence in neighboring cells, disrupt immune function, remodel the extracellular matrix, and potentially promote cancer progression [5]. These SASP factors serve as valuable biomarkers for aging and age-related diseases and are crucial for pre-clinical quality assessment of MSC-based therapeutic products. Variability in donor age, in vitro culture practices, and the presence of senescent cells are significant contributors to the inconsistent outcomes observed in MSC clinical trials [5].

This variability is further compounded by the tissue source of the MSCs. Umbilical cord-derived MSCs (ucMSCs) are particularly promising due to their high proliferative capacity, low immunogenicity, and ease of isolation. To ensure their reliable medical application, a deep understanding of their physiological responses, including those to senescent stimuli, is essential.

Given that the therapeutic effects of MSCs are largely paracrine, a comprehensive characterization of the secretome is paramount. While early proteomic studies focused on targeted analyses, modern untargeted proteomic profiling now enables a systems-level view of the cellular response to perturbations like senescence. Therefore, the aim of this work was to employ an untargeted proteomic approach to identify senescence-associated proteins within the ucMSC secretome that may regulate aging processes and intercellular communication.

## 2. Materials and Methods

### 2.1. Cell Culture and Senescence Induction

Human umbilical cord-derived MSCs (ucMSCs) were obtained from CryoCenter LLC. Cells were cultured in αMEM medium (Gibco, Life Technologies, Carlsbad, CA, USA) supplemented with 10% fetal bovine serum (HyClone, Logan, UT, USA), 50 U/mL penicillin (PanEco, Moscow, Russia), and 50 μg/mL streptomycin (PanEco) at 37 °C in a humidified 5% CO_2_ atmosphere (Binder incubator, Sanyo, Japan). Subculturing was performed at 80–90% confluence.

To induce senescence, a stress-induced model using mitomycin C (MmC) was employed. Briefly, near-confluent (90–100%) cells were treated with 1.5 μg/mL MmC in complete growth medium for 18 h. Following two washes with PBS, the cells were maintained for 10 days without passaging to allow for the full establishment of senescence. Control cells were cultured under identical conditions without MmC exposure. The culture medium was completely refreshed on day 7 post-treatment, and conditioned medium samples for analysis were collected on day 10.

### 2.2. Senescence-Associated β-Galactosidase (SA-β-Gal) Staining

Ten days after MmC exposure, cells were reseeded at a density of 3000 cells/cm^2^. After 48 h, the cells were fixed and stained for SA-β-gal activity using a commercial histochemical staining kit (Sigma, St. Louis, MO, USA), following the manufacturer’s protocol. Stained cells were visualized and analyzed using a Nikon Eclipse TiU light microscope (Nikon, Tokyo, Japan).

### 2.3. Sample Preparation for Proteomic Analysis

Conditioned medium samples were centrifuged to remove cell debris. Proteins were then reduced with 0.1 M dithiothreitol in 8 M urea/0.1 M Tris buffer at 50 °C for 45 min and alkylated with 0.05 M iodoacetate in the dark at room temperature for 20 min. Subsequently, proteins were precipitated overnight at −20 °C by adding five volumes of acetone containing 0.1% trifluoroacetic acid. The resulting protein pellet was washed sequentially with cold acetone and 96% ethanol, with centrifugation between each wash to recover the precipitate.

The pellet was resuspended in 100 µL of 0.05 M ammonium bicarbonate buffer and digested overnight at 37 °C with a trypsin/Lys-C mix (1 µg/µL) at a 1:100 (enzyme-to-protein) ratio under constant agitation (750 rpm). The digestion was quenched by acidification with formic acid. Samples were centrifuged at 21,000× *g* for 10 min, and the supernatant was desalted using OASIS HLB µElution plates (Waters) prior to LC-MS/MS analysis.

### 2.4. LC-MS/MS Proteomic Analysis

Tryptic peptides were analyzed using a Dionex Ultimate 3000 nano-HPLC system (Thermo Fisher Scientific, Waltham, MA, USA) coupled online to a timsTOF Pro mass spectrometer (Bruker Corporation, Billerica, MA, USA). Peptide separation was performed on a reversed-phase C18 column (25 cm × 75 µm, 1.6 µm bead size; Ion Optics) at a flow rate of 400 nL/min. A 40 min linear gradient from 4% to 90% mobile phase B (0.1% formic acid in acetonitrile) in mobile phase A (0.1% formic acid in water) was used.

Mass spectrometry data were acquired using the parallel accumulation–serial fragmentation (PASEF) method. The electrospray ionization source was operated with a capillary voltage of 1500 V, an endplate offset of 500 V, and a dry heater temperature of 180 °C. MS and MS/MS data were collected over an *m*/*z* range of 100–1700 Th and an ion mobility range of 0.60–1.60 Vs/cm^2^. The total cycle time was 1.88 s, including 10 PASEF MS/MS scans.

### 2.5. ELISA and Quantitative PCR Analysis

To characterize the paracrine activity of MSCs, CM was collected, centrifuged at 2500× *g* to remove cell debris, and stored at −80 °C until the measurements. IL-6 concentrations in MSC conditioned medium were evaluated using Human IL-6 ELISA Set (BD), according to the manufacturer’s instructions.

To evaluate gene expression, total RNA was extracted with ExtractRNA Reagent (Evrogen, Moscow, Russia) and purified using the phenol/chloroform technique. The quality and concentration of RNA samples were estimated by using a Nanodrop ND-2000c (Thermo Scientific, Waltham, MA, USA). Ambion DNase I (RNase-free) (Thermo Fisher Scientific, Waltham, MA, USA) was used for genomic DNA degradation. Reverse transcription was performed using the MMLV RT Kit (Evrogene, Moscow, Russia) according to the manufacturer’s protocol. Expression of the genes *IL6*, *PLAU*, *CCL2*, and *MMP1* was analyzed using Qiagen primers (Qiagen, Hilden, Germany). The expression levels of two housekeeping genes *HPRT* and *RPLP0*) were used for reference. The cDNA was mixed with qPCRmix-HS SYBR (Evrogene, Moscow, Russia) and added to 96-well plates according to the manufacturer’s protocol. The expression levels of two housekeeping genes HPRT and RPLP0) were used for reference. Polymerase chain reaction was performed using the Mx300P system (Stratagene, San Diego, CA, USA). Normalized gene expression was calculated with the 2^−ΔΔCt^ method.

### 2.6. Data Processing and Bioinformatics

Raw LC-MS/MS data were processed for peptide and protein identification using PEAKS Studio 8.5. The search parameters were: precursor mass error tolerance of 20 ppm, fragment mass error tolerance of 0.03 Da, enzyme specificity set to trypsin with up to 3 missed cleavages, fixed modification of carbamidomethylation (C), and variable modifications of oxidation (M) and N-terminal acetylation. Protein identifications were filtered to a false discovery rate (FDR) of ≤1%.

Semi-quantitative analysis was based on the peak intensity of identified peptides. Functional annotation of the identified proteins, including molecular functions and biological processes, was performed using the DAVID (https://davidbioinformatics.nih.gov/tools.jsp (accessed on 11 August 2025)) and STRING (https://string-db.org (accessed on 11 August 2025)) databases. Protein sequences and annotations were retrieved from UniProt (https://www.uniprot.org/ (accessed on 13 August 2025)).

GenAI tool DeepSeek-V3.2. (https://chat.deepseek.com (accessed on 10 November 2025)) was employed for text editing, including grammar, spelling, punctuation, formatting, and language improvement.

This study was approved by the Bioethics Committee of the Russian Research Center—Institute of Biomedical Problems of the Russian Academy of Sciences (Permission No. 550/MSK/22/07/20).

## 3. Results

### 3.1. Induction of Cellular Senescence

Cellular senescence was induced in MSCs using the DNA alkylating agent mitomycin C (MmC). MmC inhibits proliferation and promotes key senescence markers, including increased senescence-associated β-galactosidase (SA-β-gal) activity, elevated reactive oxygen species (ROS), expression of the senescence-associated secretory phenotype (SASP), and upregulation of p21 and p53 [6]. This model yields a senescent phenotype comparable to replicative senescence [7]. Following a single 18 h MmC treatment, cells were maintained in culture for 10 days without passaging to allow for the full establishment of the senescent state.

**Figure 1:**
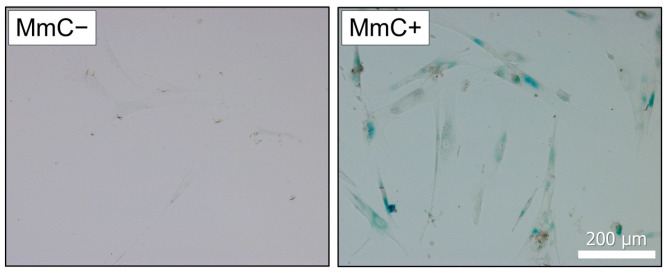
SA-β-gal staining of control and senescent MSCs. Cells were stained 48 h after passaging, which was performed 10 days after mitomycin C (MmC) treatment. Images are from a representative experiment.

Senescence induction was confirmed by SA-β-gal staining (Figure 1). MmC-treated cultures exhibited SA-β-gal activity in nearly 100% of cells, whereas no positive staining was detected in untreated control cultures.

### 3.2. Proteomic Profiling of the Senescent MSC Secretome

Panoramic proteomic analysis of mesenchymal stromal cell (MSC) conditioned medium (CM) identified over 600 proteins. Specifically, 447 proteins were detected in the CM from control MSCs, compared to 544 proteins in the CM from senescent MSCs induced by mitomycin C (MmC).

To distinguish proteins secreted by MSCs from those derived from the fetal bovine serum (FBS) in the culture medium, we also analyzed the serum-containing medium itself. This step was critical, as many bovine proteins are homologous to human proteins and can confound the analysis. Culturing MSCs in serum-free medium (αMEM without FBS, including growth factors) was not a viable alternative, as it induces significant stress, alters morphology, promotes senescence, and impairs differentiation capacity, thereby obscuring the specific effects of MmC treatment. It is interesting to note that some studies have shown that even the use of commercial serum-free media supplemented with growth factors, employed in medical protocols, can alter cell properties, including increased senescence [8]. Proteins identified in the FBS-containing medium were excluded from subsequent analysis if their abundance was higher in the medium control than in the CM samples.

**Figure 2:**
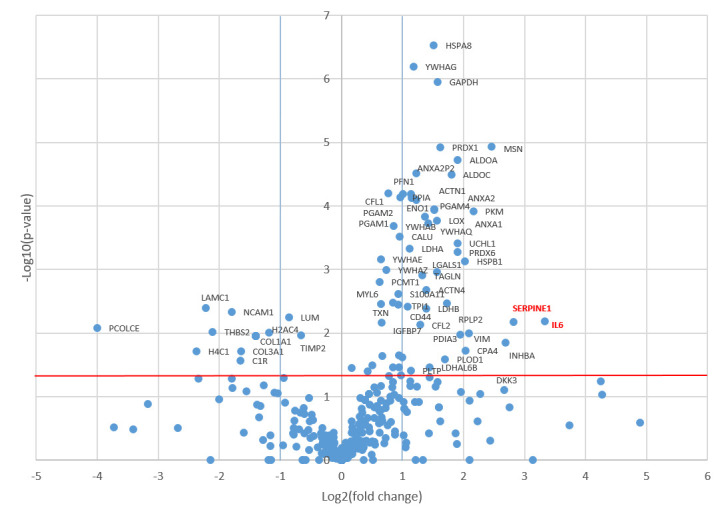
Proteomic alterations in the senescent MSC secretome. The volcano plot displays proteins identified in the conditioned medium of mitomycin C-treated senescent MSCs versus untreated controls. Proteins with significantly altered abundance according to *t*-test (*p*-value < 0.05, indicated by the red horizontal line) and a fold-change greater than 2 (log_2_(fold change) > 1 or <−1, indicated by blue vertical lines) are highlighted. Right quadrant: upregulated proteins; Left quadrant: downregulated proteins.

**Figure 3:**
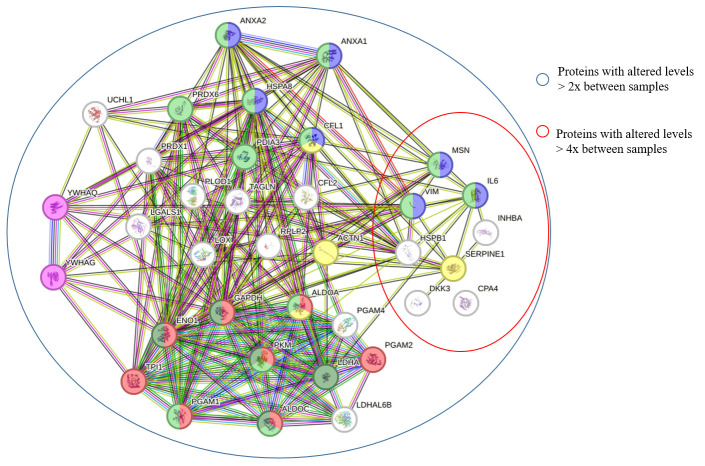
Protein–protein interaction network of upregulated senescent MSC secretome proteins. The network illustrates interactions between proteins significantly upregulated (*p*-value < 0.05 and fold-change > 2) in the secretome of senescent MSCs. Proteins are color-coded based on the pathways they participate in: glycolysis (red), senescent glycolysis (dark green), immune system (light green), hemostasis (yellow), cyclin complex inactivation (purple), and interleukin signaling (blue).

Comparative analysis using the Mann–Whitney test revealed 57 proteins that were significantly upregulated in the CM of senescent MSCs (Figure 2). Of these, 38 proteins increased more than twofold and 8 proteins more than fourfold (Figure 3). The most strongly upregulated proteins were interleukin-6 (IL-6) and plasminogen activator inhibitor-1 (PAI-1, SERPINE1), which exhibited 10-fold and 7-fold increases, respectively. Consistent with this, the volcano plot identified IL-6 and PAI-1 as the most significantly altered proteins, displaying the highest log_2_(fold change) values with a high level of statistical significance (*p* < 0.05).

### 3.3. Functional Annotation of the Senescent MSC Secretome

**Figure 4:**
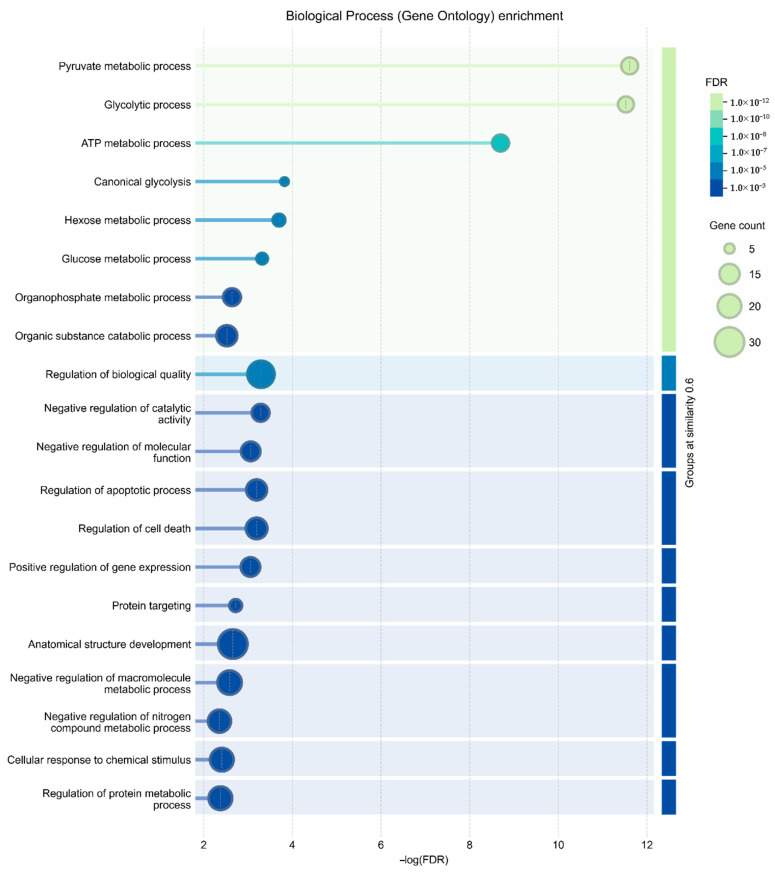
Gene Ontology (GO) enrichment analysis of biological processes. The bar chart displays the 20 most significantly enriched biological processes among proteins upregulated in the secretome of senescent MSCs compared to controls. Enrichment significance is shown as −log_10_(FDR).

**Figure 5:**
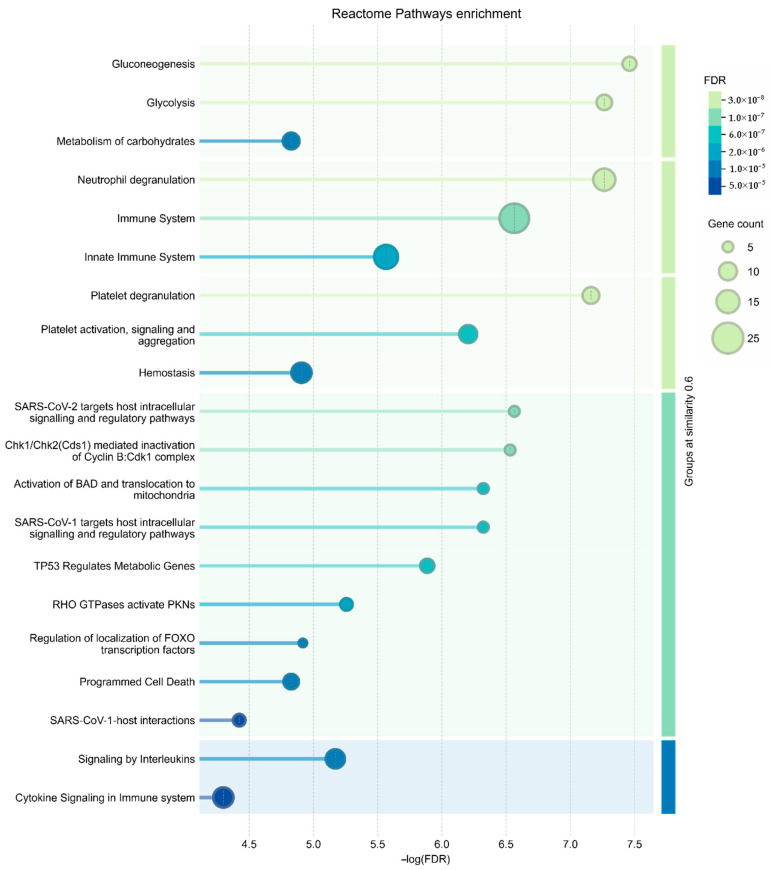
Reactome pathway enrichment analysis. The chart displays the top 20 significantly enriched pathways from the Reactome database involving proteins upregulated in the secretome of senescent MSCs. Enrichment is plotted as −log_10_(FDR).

**Figure 6:**
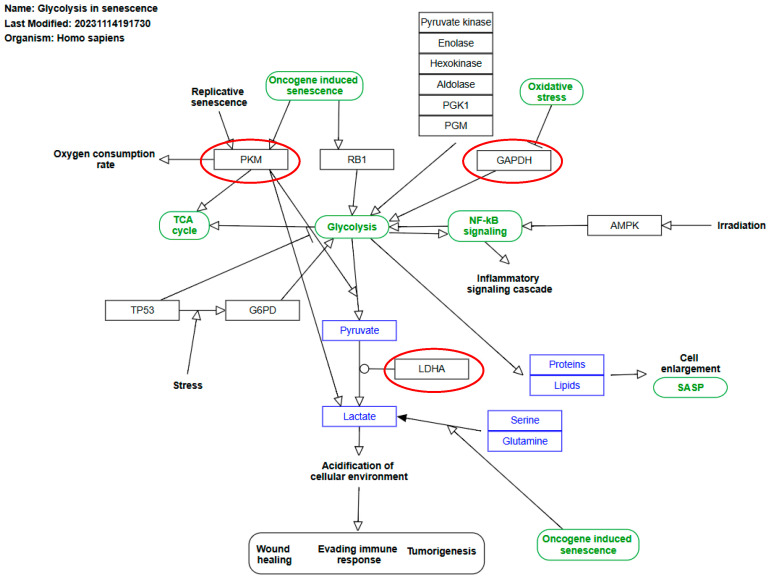
Schematic of the “Glycolysis in Senescence” pathway from WikiPathways. Proteins significantly upregulated in the conditioned medium (CM) of senescent MSCs are highlighted in red.

To investigate the functional relationships among proteins upregulated in senescent MSCs, a protein–protein interaction (PPI) network was constructed using the STRING database. The network revealed a high degree of connectivity among these secreted factors. Gene Ontology (GO) analysis identified the 20 most significantly enriched biological processes (Figure 4) and pathways (Figure 5). A prominent finding was the strong association of the senescent secretome with glycolysis. Multiple glycolysis-related proteins were upregulated (TPI1, PGAM2, LDHAL6B, PKM, PGAM1, ALDOC, GAPDH, PGAM4, LDHA, ENO1, ALDOA), as visualized in the PPI network (Figure 3). This was further corroborated by the significant enrichment of the “Glycolysis in Senescence” WikiPathways entry, which included core enzymes such as GAPDH, LDHA, ENO1, PKM, and ALDOC (Figure 6).

A robust body of evidence links the activation of glycolysis to various aging phenotypes. Metabolic profiling has consistently shown a shift toward a glycolytic state in replicative senescence, albeit one that is less energetically efficient [9]. This metabolic reprogramming is thought to support several senescence-associated processes, including increased biosynthetic demands, compensation for mitochondrial dysfunction, maintenance of redox homeostasis, and activation of specific signaling pathways [10].

Glycolysis directly fuels the senescent state through multiple mechanisms. It can activate NF-κB signaling, driving the pro-inflammatory cascades characteristic of senescence. Furthermore, glycolytic activation increases the production of lactate and pyruvate, driven by enzymes such as lactate dehydrogenase (LDHA) and pyruvate kinase (PKM). Notably, PKM activation during replicative senescence can paradoxically increase TCA cycle activity and oxygen consumption [11], while also contributing to elevated lactate production [9]. The concurrent rise in LDHA further amplifies lactate levels, which have been implicated in age-associated pathologies like immune evasion and oncogenesis [12].

The tumor suppressor p53, a central mediator of senescence, plays a complex and context-dependent role in regulating this metabolic shift. While some studies indicate p53 can suppress glycolysis, it has also been shown to exert a positive effect under stress by activating glucose-6-phosphate dehydrogenase (G6PD) [13]. Therefore, p53 is considered to play a regulatory role in glycolysis and is of interest in the context of aging. In our model, where senescence was induced by the DNA-damaging agent mitomycin C—a known activator of the p53 pathway [14]—the observed upregulation of glycolytic proteins in the secretome is consistent with p53-mediated metabolic reprogramming.

Our data strongly support this paradigm. We observed a coordinated increase (more than twofold) in the expression of glycolytic proteins. Most strikingly, pyruvate kinase PKM was upregulated 4.5-fold. The critical role of PKM2 in senescence is underscored by studies showing that its suppression in aged chondrocytes reduces established senescence markers (p16 and SA-β-gal) and promotes a rejuvenated phenotype [15]. This confirms pyruvate kinase as a pivotal regulator in the aging process and validates our proteomic findings.

Following glycolysis, the most significantly enriched biological process was “Regulation of biological quality” (SERPINE1, LOX, INHBA, HSPB1, PRDX1, CFL2, YWHAG, PRDX6, ANXA2, MSN, TXN, ANXA1, GAPDH, IL6, CFL1, DKK3, HSPA8, VIM, ALDOA). Many of these proteins are involved in the following processes, as highlighted by the String web-resource: processes of negative regulation of catalytic activity and molecular functions, regulation of apoptosis and cell death, as well as positive regulation of gene expression. The latter is particularly relevant, as proteins like IL-6 and PAI-1 (SERPINE1 gene) are established inducers of senescence and may initiate broader signaling cascades in response to stress. Notably, almost all proteins within this group were upregulated by at least twofold in the senescent MSC secretome, underscoring their central role in the cellular response to senescence.

Pathway analysis via the Reactome database further contextualized these findings. While glycolysis constituted the most enriched pathway (Group I), the subsequent categories were neutrophil degranulation (Group II) including immune system proteins (ANXA2, ANXA1, HSPA8, PRDX6, PDIA3, CFL1, MSN VIM, IL6, ALDOA, ALDOC, PGAM1, PKM), and platelet degranulation (Group III), including hemostasis proteins (SERPINE1, CFL1, ACTN1, ALDOA). This aligns with known MSC biology, as MSCs can modulate coagulation through various mechanisms, including the expression of tissue factors and the promotion of platelet activation [16]. Furthermore, our results are consistent with prior proteomic studies showing that serial passaging of MSCs increases the secretion of pro-coagulant proteins, suggesting a senescence-associated elevation in thrombotic risk [17].

Our proteomic analysis identified several key proteins in the senescent MSC secretome with critical implications for hemostasis and thrombosis. The most prominent was Plasminogen Activator Inhibitor-1 (PAI-1), encoded by the SERPINE1 gene. PAI-1 is a well-established regulator of vascular pathologies, including arterial thrombosis and perivascular fibrosis [18,19]. Supporting its pathogenic role, transgenic mice overexpressing PAI-1 develop age-related atherosclerosis, whereas PAI-1-deficient animals are protected from experimental vascular disease [19]. PAI-1 may represent a classic example of antagonistic pleiotropy, providing benefits early in life but becoming detrimental with age. Consequently, the suppression of PAI-1 is now being investigated as a therapeutic strategy for age-associated conditions like thrombosis, fibrosis, and diabetes [20].

Furthermore, we observed an increase in ACTN1, a protein recently identified alongside coagulation factor F5 as a novel biomarker linked to coagulation and the immune microenvironment in ischemic stroke [21]. The co-upregulation of PAI-1 and ACTN1 in our model suggests that senescent MSCs adopt a prothrombotic phenotype. This finding underscores the critical importance of stringent quality control—including monitoring culture passages and conducting comprehensive secretome profiling—for MSCs intended for therapeutic use to mitigate the risk of adverse coagulation events.

Beyond hemostasis, pathway analysis revealed a significant group of proteins involved in TP53 regulation, cell cycle arrest, and programmed cell death (Group IV). This group included multiple 14-3-3 protein isoforms (YWHAB, YWHAE, YWHAG, YWHAQ, YWHAZ), which are key mediators of the DNA damage response. The 14-3-3 proteins enforce cell cycle arrest by binding to and sequestering Cdc25C in the cytoplasm, preventing it from activating the nuclear Cyclin B-Cdk1 complex necessary for the G2/M transition [22]. The elevated levels of these proteins in our study provide direct molecular evidence for the irreversible proliferation arrest characteristic of MmC-induced senescence.

Finally, a fifth group of pathways was enriched for interleukin signaling (Group V), involving proteins encoded by IL6, ANXA2, ANXA1, HSPA8, CFL1, MSN, and VIM. Interleukins are potent signaling molecules with pleiotropic effects on tissue repair, hematopoiesis, and inflammation. The significant presence of these factors in the senescent secretome highlights their potential to drive both beneficial and detrimental processes. Therefore, proteins within this group warrant particular attention when assessing the safety and efficacy of MSC-derived conditioned medium for therapeutic applications.

### 3.4. Analysis of Proteins Downregulated in the Senescent MSC Secretome

Proteomic analysis also identified proteins significantly downregulated following stress-induced senescence (Figure 2). This group was notably enriched for structural and regulatory components of the extracellular matrix (ECM), including collagens (COL1A1, COL3A1), laminin subunit gamma-1 (LAMC1), and lumican (LUM). Also decreased were key ECM-stabilizing factors such as the metalloproteinase inhibitor TIMP2, the intercellular interaction regulator thrombospondin-2 (THBS2), the fibrillogenesis regulator decorin (DCN), and procollagen C-endopeptidase enhancer 1 (PCOLCE). The latter is critical for the proteolytic maturation of type I procollagen into stable, triple-helical fibrils.

Furthermore, the secretome of control (“young”) MSCs contained elevated levels of core histones (H2A, H4), suggesting the active release of nuclear proteins associated with DNA replication, transcription, and repair processes, which appears to be suppressed in senescence.

### 3.5. ELISA and qPCR

**Figure 7:**
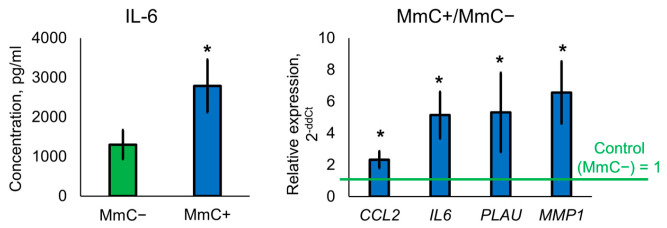
ELISA and qPCR analysis. Samples were analyzed 10 days after MmC treatment. Data are shown as mean ± SD; *n* ≥ 4, * *p* < 0.05. MmC—mitomycin C.

The results of the ELISA and qPCR fully confirmed the data obtained using proteomic methods in samples of cells subjected to stress-induced senescence using MmC (Figure 7). A significant increase in the level of the pro-inflammatory cytokine IL-6 was observed in the samples. At the same time, overexpression of mRNA for several genes (*IL6*, *PLAU*, *CCL2*, *MMP1*) was detected. MMP1 is a component of the SASP and can degrade collagen and other extracellular matrix components, leading to changes in the tissue microenvironment that can induce senescence.

The signaling pathway involving the urokinase plasminogen activator (PLAU), its receptor (uPAR), and its primary inhibitor, serpin-1 (PAI-1), plays a central role in the establishment and maintenance of the cellular senescence phenotype. The combined analysis of proteomic and transcriptomic data revealed a coordinated activation of key molecular programs characteristic of the MSC senescent phenotype. In addition to the expected increase in the secretion of canonical SASP factors (IL-6, PAI-1), qPCR analysis confirmed a significant upregulation of *IL6* and *CCL2* (MCP-1) gene expression, indicating sustained activation of the core pro-inflammatory response mediated by NF-κB/JAK-STAT pathways. Concurrently, the elevated expression of PLAU (uPA) and MMP1 genes, coupled with high levels of the PAI-1 protein, reflects a profound dysregulation of proteolytic and fibrinolytic systems, shifting the balance toward pathological extracellular matrix remodeling and a pro-thrombotic state. Thus, mitomycin C-induced senescence initiates a comprehensive transcriptional-secretory reprogramming that establishes a pro-inflammatory and pro-fibrotic microenvironment.

Thus, the combination of proteomic screening, ELISA, and qPCR analysis demonstrated a consistent enhancement of the inflammatory response at both the translational and transcriptional regulation levels under conditions of stress-induced cellular senescence caused by MmC.

## 4. Discussion

This study characterized the proteomic profile of the secretome from human umbilical cord MSCs (ucMSCs) undergoing stress-induced senescence via mitomycin C (MmC) treatment. Senescence was confirmed ten days post-treatment by a significant increase in SA-β-gal activity. Subsequent proteomic analysis revealed a profound remodeling of the ucMSC secretome, marked by the upregulation of proteins involved in biological processes, such as glycolysis, negative regulation of catalytic activity and molecular functions, regulation of apoptosis and cell death, as well as positive regulation of gene expression. Proteins with elevated levels were related to pathways such as glycolysis, neutrophil degranulation and immune system, platelet degranulation and hemostasis, cell cycle regulation, and signaling by interleukins.

Our proteomic analysis employed a stringent filtering step to distinguish human MSC-secreted proteins from bovine serum background, which, while ensuring specificity, may have led to the exclusion of some low-abundance human proteins—a recognized limitation of studies using serum-supplemented media.

Among the upregulated proteins, we identified several established components of the senescence-associated secretory phenotype (SASP), including IL-6, SERPINE1 (PAI-1), IGFBP7, and TXN (Figure 3). IL-6, a pleiotropic cytokine and one of the most highly expressed SASP factors, can reinforce the senescent state and is linked to increased age-related mortality [23,24].

A central finding of our study is the pronounced upregulation of PAI-1, encoded by the SERPINE1 gene and described above in the context of thrombosis regulation. PAI-1 is a consistently robust marker across diverse senescence models [25,26], and its increase here is mechanistically consistent with the activation of the p53 pathway by MmC-induced DNA damage. Our data corroborate previous work demonstrating PAI-1 accumulation in the extracellular matrix following MmC exposure [27]. Critically, PAI-1 is not merely a passive biomarker but an active driver of the senescent phenotype; it is both necessary and sufficient for the induction of replicative senescence and can independently initiate cellular senescence [28,29], positioning it as a master regulator of the aging program [30].

Consequently, PAI-1 functions as a pivotal node in aging, exerting both autocrine and paracrine effects. Elevated PAI-1 expression can promote a self-sustaining, positive feedback loop that amplifies its own production and that of other senescence regulators. Given its well-established role in promoting thrombosis and other age-related pathologies, PAI-1 emerges from our data as a possible candidate for pharmacological intervention to counteract cellular senescence and mitigate age-associated diseases [20].

Our analysis also confirmed the upregulation of other critical SASP components, including insulin-like growth factor binding protein 7 (IGFBP7). This protein, which regulates the bioavailability of insulin-like growth factors, can directly induce a senescent state in recipient cells. Conversely, neutralizing antibodies against IGFBP7 can attenuate SASP-mediated senescence, underscoring its active role in this process [31].

We also observed a significant increase in secreted proteins with antioxidant functions, such as thioredoxin (TXN) and peroxiredoxins (PRDX1, PRDX6). While their secretion is a hallmark of the senescent state, often in response to elevated ROS, these proteins primarily serve a protective role. TXN, a redox-active protein induced by diverse cellular stresses, has demonstrated therapeutic efficacy in various inflammatory disease models, including viral pneumonia, acute lung injury, gastric injury, dermatitis, and others [32]. Similarly, PRDX6 from mesenchymal stem cell-derived extracellular vesicles can suppress oxidative stress in chondrocytes [33]. Thus, the secretion of TXN, PRDX1, and PRDX6 may represent a compensatory mechanism to mitigate oxidative damage and slow the progression of senescence in neighboring cells.

This theme of a dualistic secretome—mixing inflammatory SASP factors with protective elements—was further exemplified by the upregulation of annexins ANXA1 and ANXA2 (Figure 3). Although primarily intracellular proteins, they can be secreted and exert anti-inflammatory, pro-fibrinolytic, and anti-coagulant effects in the extracellular space [34]. Notably, recombinant ANXA2 has been shown to enhance autophagy, reduce ROS, and delay cellular senescence [35].

Similarly, the small heat shock protein HSPB1, which increased more than fourfold in the senescent secretome, functions as a molecular chaperone. Its primary role in aging contexts is to protect against proteotoxicity, particularly under conditions of oxidative stress [36], further highlighting the complex nature of the senescent MSC secretome.

Our analysis also identified a group of proteins upregulated more than fourfold, including INHBA, VIM, and DKK3 (Figure 3), each with significant implications for the senescent phenotype.

The level of inhibin beta A subunit (INHBA) increased 6.5-fold in senescent cells. As a component of activin and inhibin in the TGF-β superfamily, INHBA has complex roles in growth regulation. Its age-associated increase in human epidermis contributes to thinning and reduced progenitor cell proliferation [37]. Furthermore, INHBA has been identified as a novel regulator of cellular senescence and immune evasion in cancer [38], highlighting its potential broader role in aging pathologies.

We also observed a greater than fourfold increase in extracellular vimentin (VIM). Senescent cells are known to overexpress this intermediate filament protein, which undergoes a structural reorganization from a loose network to tightly bundled filaments. This cytoskeletal remodeling in senescence may facilitate the release of vimentin into the extracellular space. While the function of secreted vimentin is not fully defined, it is implicated in pro-inflammatory signaling and immune activation [39], suggesting its release contributes to the altered microenvironment.

Finally, Dickkopf-related protein 3 (DKK3), a secreted inhibitor of the Wnt/β-catenin pathway, was also upregulated more than fourfold. This finding is consistent with other studies of the senescent MSC secretome [17]. Since canonical Wnt signaling is a critical regulator of stem cell self-renewal and pluripotency [40], the sustained secretion of DKK3 likely serves to irreversibly suppress these core MSC functions, reinforcing the senescent growth arrest.

Although senescent mesenchymal stromal cells are excluded from therapeutic products, their secretome highlights a critical link between inflammation and thrombosis—a key risk in MSC therapy. Clinically, even high-quality MSC products in controlled trials have been associated with mild to severe thromboembolic events. A primary mediator is the expression of tissue factor (TF/CD142), a potent procoagulant [41]. However, some MSC products exhibit procoagulant activity that persists despite TF blockade. The mechanism underlying this residual activity remains unknown but may involve the inhibition of fibrinolysis by plasminogen activator inhibitor-1 (PAI-1) [42]. To address the risk of TF-independent thromboembolic complications, we propose that the characterization of clinical MSC products should include assessment of PAI-1 content. Extending this analysis to a broader preliminary screen for key pro-inflammatory and prothrombogenic SASP components would significantly improve product quality and therapeutic safety.

Our proteomic analysis of the ucMSC secretome exposed to stress-induced senescence with mitomycin C revealed significant activation of canonical SASP factors, particularly IL-6 and SERPINE1 (PAI-1). These data correlate with the results of independent studies using alternative models of senescence. Samsonraj et al. showed that oxidative stress induced by H_2_O_2_ causes a similar increase in IL-6 and SERPINE1 secretion in bone marrow-derived MSCs [43]. In parallel, a comparative analysis of BM-MSCs and iMSCs during replicative senescence also confirmed a steady increase in the levels of these cytokines, with SERPINE1 expression being higher in BM-MSCs, consistent with their more pronounced senescent phenotype [44]. The study, similar to our data, identified protective components such as thioredoxin (TXN) and peroxiredoxin-1 (PRDX1) in the senescent secretome, supporting the concept of the complex and dual nature of the SASP. At the same time, as demonstrated by Samsonraj et al., certain inducers (e.g., ionizing radiation) are capable of imparting unique, inducer-specific features to the SASP [43]. Thus, the present study contributes to defining both the conserved “core” of the SASP and its variable elements, substantiating the fundamental value of detailed proteomic profiling—even within a single validated model of senescence—for assessing the quality of cellular products.

## 5. Conclusions

This study characterized the proteomic remodeling of the MSC secretome following stress-induced senescence by mitomycin C. We confirmed the upregulation of established senescence markers (IL-6, PAI-1, IGFBP7) and identified significant increases in less characterized proteins like INHBA, VIM, and DKK3, which also play a role in senescence processes but have not yet received much attention due to the less consistent results of studies on aging markers in various cell cultures. Overall, stress-induced senescence affected pathways of glycolysis, immune system function, hemostasis, cell death, and cell cycle regulation, and interleukin signaling. These pathways determine the properties of MSCs that are revealed during senescence—proliferation arrest, increased lactate, and the formation of a prothrombogenic and pro-inflammatory environment—which negatively impacts the MSC microenvironment and can induce senescence in neighboring cells.

Notably, the senescent secretome exhibited a dual nature. Alongside detrimental factors, we detected protective proteins, including annexins, proteins with antioxidant activity, and heat shock proteins, which may mitigate oxidative stress and inflammation. These findings highlight the complexity of the senescence-associated secretory phenotype (SASP) and underscore the necessity of comprehensive secretome profiling in various aging models. Such detailed characterization is crucial for harnessing the therapeutic potential of MSCs while mitigating the risks associated with senescent cells in regenerative medicine.

## Data Availability

The original contributions presented in this study are included in the article. Further inquiries can be directed to the corresponding author.
